# Exploring the Relationship Among Teacher Emotional Intelligence, Work Engagement, Teacher Self-Efficacy, and Student Academic Achievement: A Moderated Mediation Model

**DOI:** 10.3389/fpsyg.2021.810559

**Published:** 2022-01-03

**Authors:** Li Wang

**Affiliations:** School of Chinese Literature and Media, Hubei University of Arts and Science, Xiangyang, China

**Keywords:** teacher emotional intelligence, work engagement, teacher self-efficacy, student academic achievement, moderated mediation effect

## Abstract

In recent years, many studies have been done to identify the factors that affect teacher emotions at schools. However, the empirical evidence on how teachers’ emotions influence students’ outcomes and performance is not extensive. Against this background, this study explored the correlation between teacher EI and student academic achievement and possible mechanisms may lie in this relationship. A sample of 365 Chinese teachers from 25 public middle schools participated in this study by completing measurements of teacher EI, teacher work engagement, and teacher self-efficacy. The student academic achievement was assessed by the grades of the previous term (February to June 2020) reported by the students. The results indicated that teacher work engagement partially mediated the path from teacher EI and student academic achievement. Moderated mediation further showed that teachers with high self-efficacy had a more significant positive impact on the relationship between teacher work engagement and student academic achievement than teachers with low self-efficacy. The limitations of this study were also discussed.

## Introduction

Emotions are complex psycho-physiological processes triggered by subjectively important events in an individual’s life (Eisma and Stroebe, 2021). They have been studied by psychologists for more than a century (Berridge, 2018). Some researchers claimed that teaching is an emotional process, in which teachers manage, scrutinize, and control their feelings to achieve teaching effectiveness, to inspire students, and to create a positive environment for learning (Schonert-Reichl, 2017). The study of teacher emotions has increased remarkably since the mid-1990s, which has led educators to pay more attention to the relevance of emotional intelligence to their work (Yin, 2012; Maamari and Majdalani, 2019).

Emotional intelligence (EI), a term coined by Salovey and Mayer (1990), is usually concerned with how people perceive, regulate, and express their own personal emotions as well as other people’s feelings (Maamari and Majdalani, 2019). The current understanding of emotional intelligence in the academic domain mainly consists of two aspects: ability emotional intelligence (ability EI) and trait emotional intelligence (trait EI). The first model conceptualizes EI as a form of a cognitive ability that involves understanding and distinguishing emotional signals and information, while the second sees EI as a personality trait that is related to typical behavior (Bar-On and Parker, 2000; Lu et al., 2016). These two different models have led to the distinct measurement methods and underlying empirical bases of EI (Davis and Nichols, 2016). Considerable research on EI has found that high EI is associated with positive life outcomes, such as developing positive social relationships, identifying others’ emotional states, adjusting to others’ perspectives, enhancing communication, and managing behavior (Miao et al., 2017). In addition, low levels of EI are seen as a tendency toward self-destructive and deviant behaviors (Curci et al., 2014; Davis and Nichols, 2016), such as taking illegal drugs and consuming excessive amounts of alcohol, having poor relationships with friends, being absent from school without authorizations and expelled from school, and having feelings of depression (Brackett et al., 2004; Davis and Humphrey, 2014).

In the realm of education, scientific literature has also manifested the association between EI and successful outcomes. For example, Palomera et al. (2008) found that high levels of emotional intelligence in teachers play a significant role in teaching. Similarly, studies by Extremera and Fernández-Berrocal (2013) showed that students with a high degree of emotional intelligence are more likely to have better academic scores, psychological adjustment, social relationships, and social behavior. While a large body of research examining the relationship between teacher EI and their educational work, or between student EI and their various outcomes, empirical evidence on how teacher emotions affect student outcomes and performance is rare. Based on Frenzel’s (2014) reciprocal model of the antecedents and consequences of teachers’ emotions, we wanted to know if teachers’ high emotional intelligence was somehow beneficial to their students. Therefore, the present study, using a structural equation modeling approach, aims to explore the correlation between teacher EI and student academic achievement and possible mechanisms may lie in this relationship.

## Materials and Methods

### Participants and Procedure

The sample was composed of 365 teachers from 25 public middle schools of Hubei province in the center of China. The 365 participants included 198 males (54%) and 167 females (46%). 37% of the sample were in grade seventh, 35% were in grade eighth, and 28% were in grade ninth. The average age of the participants was 38.4 years (SD = 5.3 years). The teachers’ average experience in terms of years was 6.7(SD = 2.1). There were about 72% of the participants held a bachelor’s degree, 24% held a master’s degree, and 4% held a doctoral degree. Prior to the investigation, the researcher contacted the school administrators by emails or phones, and asked them to invite their teachers to participate this research. The questionnaires were originally designed in English. The researchers translated and back-translated the English language of the questionnaires, and then conducted data collection with the Chinese version. The questionnaires were accompanied by a covering letter explaining the purpose and process of the project. The participation in this survey was voluntary. Participants could withdraw at any time during the survey, and all of their answers were confidential and anonymous.

### Measures

The teacher EI was assessed with the Wong and Law’s Emotional Intelligence Scale (WLEIS: Wong and Law, 2002). It is a 16-item self-report EI measure that comprises 4 sub-scales: self-emotion appraisal (SEA; 4 items—“I have good understanding of my own emotions”), others’ emotion appraisal (OEA; 4 items—“I am a good observer of others’ emotions”), use of emotion (UOE; 4 items—“always tell myself I am a competent person”), and regulation of emotion (ROE; 4 items—“I am quite capable of controlling my own emotions”). Participants’ response was recorded using a 7-point Likert type scale from 1 = “totally disagree” to 7 = “totally agree.” The Cronbach’s alpha of teacher EI for this study was 0.843.

The teacher work engagement was assessed using the ETS (Klassen et al., 2013). It is consisted of 16 items. These items are distributed in four dimensions: cognitive engagement (CE; 4 items), example: “while teaching, I work with intensity”; emotional engagement (EE; 4 items) and example: “I am excited about teaching; social engagement with the students” (SEC; 4 items), example: “In class, I care about the problems of my students”; and social engagement with the colleagues (SES; 4 items), example: “At school, I value the relationships I build with my colleagues.” The measure is rated on seven-point Likert scales ranging from 1 = “never” to 7 = “always.” In the current sample, the Cronbach’s alpha of teacher work engagement was 0.893.

The teacher self-efficacy is assessed with TSES (short form), developed by Tschannen-Moran and Woolfolk Hoy (2001). It is a 12-item measure that comprises 3 sub-scales: efficacy in student engagement (4 items—“How much can you do to motivate students who show low interest in schoolwork?”), efficacy in instructional strategies (4 items—“To what extent can you craft good questions for your students?”), and efficacy in classroom management (4 items—“How much can you do to motivate students who show low interest in schoolwork?”). The measure is assessed by a 9-point Likert scale from 1 to 9, ranging from “none” in the “a great deal.” The Cronbach’s alpha coefficient of TSES was 0.866.

The students’ academic achievement was assessed by the grades of the previous term (February to June 2020) reported by the students. A class average score was assigned to the corresponding teacher participant. It was calculated based on one mandatory subject in the Chinese education curriculum: mathematics. Students’ grades were ranged from 1 (“insufficient”) to 5 (“outstanding”) so higher scores indicate better academic performance.

### Data Analysis

Structural equation modeling (SEM) was utilized to test our hypotheses. The software used to perform SEM analyses was Mplus version 7.4 (Muthén and Muthén, 2015). First, we conducted a total effect analysis to test the relationship between teacher EI and student academic achievement (H1). Then, we conducted a mediation analysis to test the mediating effect of teacher work engagement on the relationship between teacher EI and student academic achievement (H2). Finally, we performed a moderated mediation model to test the moderating effect of teacher self-efficacy on the relationship between teacher work engagement and student academic achievement (H3), as well as on the mediating effect of teacher work engagement abovementioned.

Particularly, we used the Latent Moderated Structural Equations (LMS) method to construct the latent interaction term of teacher work engagement and self-efficacy (Cheung and Lau, 2017). Moreover, we employed the bootstrapping method to determine the significance of the mediating effect of teacher work engagement and the moderated mediating effect, because both effects involve the product of two path coefficients (Zhao et al., 2010; Hayes, 2015). Specifically, we used 1,000 bootstrapped resamples to compute the 95% confidence intervals (CI) of the mediating effect and the moderated mediating effect. The focused effects can be determined as significant if the 95% CI did not contain zero (Preacher and Hayes, 2008).

## Hypothesis Development

### Teacher EI and Student Academic Achievement

According to Bar-On (2010), EI is a component of positive psychology that has significant implications for human performance, wellbeing, and subjective wellbeing. In the context of education, teacher EI is an important personal resource when teachers are faced with the demands of their profession (Valente et al., 2020). EI has been categorized by Chan (2004) into four dimensions: emotional appraisal, positive regulation, empathic sensitivity, and positive utilization. Emotional appraisal refers to the assessment of self-emotions (e.g., knowing the reasons for mood changes), positive regulation refers to the regulation of self-emotions (e.g., expecting good things to happen), empathic sensitivity refers to the recognition of others’ emotions (e.g., recognizing emotions from facial expressions), and positive utilization refers to the use of emotions (e.g., solving problems in positive emotions).

Reviewing the previous literature on teacher EI, the majority of studies focuses primarily on the impact of teacher EI on various teacher outcomes. For example, some researchers have explored the relationship between teacher EI and self-efficacy (e.g., Moafian and Ghanizadeh, 2009). In the study of Singh and Jha (2012), they pointed out that teachers’ EI was highly relevant to their efficacy and to improve their performance. Similarly, Wu et al. (2019) found that teachers with higher EI tended to exhibit a higher level of self-efficacy. In their study, the participants demonstrated a greater motivation to teach and fewer intentions to quit the profession. Teacher EI and “burnout” have also been explored. Overall, empirical findings have shown that teachers with high scores in the highest-level dimension of EI show lower levels of exhaustion or burnout (e.g., Platsidou, 2010; Pishghadam and Sahebjam, 2012). Additionally, some studies have examined the relationship between teacher EI and job commitment. According to these studies, EI has a positive impact on teachers’ energy, focus, and persistence (e.g., Naderi Anari, 2012 Mérida-López et al., 2017).

While a large body of research indicates that teacher EI is positively correlated with various teacher outcomes, studies on the association between teacher EI and student outcomes are rare and have yielded mixed results. For example, Curci et al. (2014) argued that teacher EI contributes to student achievement by enhancing students’ perceived competence and self-esteem. Contrary to Curci et al. (2014), Koifman (1998) found no link between teacher EI and student achievement. Against this background, future research involving these two variables is necessary to be done.

Academic achievement refers to the educational outcomes of a person at educational institutions (Cheng et al., 2019). Educational institutions are not just places where knowledge is imparted, but places where educators inspire and support students (Welmilla, 2020). Modern educators are supposed to control their emotions and, equally important, establish good interactions and connections with students when providing effective instruction. Teachers with high EI levels tend to be more concerned about their students (Alam and Ahmad, 2018). They can better perceive the needs of students and respond to those needs positively. According to Welmilla (2020), teachers with high emotional intelligence are good at engaging students in learning activities, which has a positive impact on student learning outcomes. Based on the empirical evidence provided in the above section, we hypothesize that as:

*H1a*: Teachers’ emotional intelligence is positively related to student academic achievement.

### Mediation Effects of Teacher Work Engagement

Teacher work engagement is a motivational concept that refers to teachers’ voluntary allocation of physical, cognitive, and emotional resources directed at the range of tasks demanded by a teaching role (Christian et al., 2011). It is a positive, enduring, work-related mindset (Schaufeli et al., 2002). According to Klassen et al. (2013), teacher work engagement includes three domains: cognitive-physical, emotional, and social. Sometimes, these three domains are incorporated into one higher-order engagement construct, in which each domain is experienced simultaneously or holistically (Klassen et al., 2013).

According to the Job demands–resources model (Bakker and Demerouti, 2017), personal resources are one of the key factors influencing work engagement. The EI, as one of the personal resources, contributes significantly to work engagement. Reviewing the previous literature, the growth of research on the correlation between EI and work engagement in school settings has been rapid (Mérida-López et al., 2017; D’Amico et al., 2020). These studies pointed out that teacher EI is strongly related to all three work engagement dimensions. For example, Mérida-López and Extremera (2017) argue that EI can help teachers reduce burnout and thus have a positive impact on teachers’ engagement in their work.

Additionally, teacher work engagement is also considered a predictor of student academic performance (Addimando, 2019). According to Basikin (2007), engaged teachers are adept at giving students a high level of attention during the learning process, developing appropriate strategies that assist them in understanding the behavior of students, creating good lesson plans, and assessing student performance effectively in the learning process. Furthermore, a teacher who is engaged in the classroom and actively involved in developing a healthy student-teacher relationship will promote students’ engagement and thereby improve students’ academic achievement (Addimando, 2019). Thus, we propose that as:

*H1b*: Teacher work engagement has a mediation effect on the relationship between teacher EI and student academic achievement.

### Moderating Effects of Teacher Self-Efficacy

Self-efficacy reflects a person’s beliefs about his or her capacities to execute specific actions required to produce a given achievement (Bandura, 1997). In the educational setting, teacher self-efficacy refers to teachers’ self-referent judgments or perceptions about their abilities to successfully complete teaching-related tasks and bring about desired outcomes of students (Klassen et al., 2011). According to the model of teacher self-efficacy structure (Tschannen-Moran and Woolfolk Hoy, 2001), teacher self-efficacy includes three aspects, which are self-efficacy for classroom management, self-efficacy for instructional strategies, and self-efficacy for student engagement. These three dimensions have high reliability, and factor analysis confirms the presence of higher-order dimensions of teacher self-efficacy in teachers’ perceived ability to perform teaching-related tasks (Perera et al., 2019).

Prior studies have revealed that efficacious teachers who believe themselves having the ability to successfully execute teaching tasks are more likely to be engaged in their work (Granziera and Perera, 2019). In other words, teachers with a strong sense of efficacy tend to be more enthusiastic and committed to their work. For instance, Skaalvik and Skaalvik (2014) discovered that higher levels of teacher self-efficacy led to greater levels of work engagement among school teachers. Longitudinal evidence conducted by Simbula et al. (2011) has also supported the view that teachers’ self-efficacy generalizes their engagement to their work. Similarly, Lu et al. (2016) revealed that the levels of teacher self-efficacy may significantly influence their persistence, commitment, and teaching behaviors in working with challenging students. Teachers’ self-efficacy has also been noted to be one of the most significant factors that affect students’ achievement (Kim and Seo, 2018). According to Tschannen-Moran and Woolfolk Hoy (2001), teachers who are confident in their ability to teach and in their ability to motivate students tend to have a greater effect on their students’ academic performance even if the students lack academic motivation. The findings of Kim and Seo (2018) are consistent with the study of Tschannen-Moran and Woolfolk Hoy (2001). They stated that teachers with high levels of self-efficacy know the importance of their teaching confidence and how their beliefs take their students toward success in academic learning. Drawing on these evidences, we propose the hypothesis as follows:

*H2*: Teacher self-efficacy moderates the positive relationship between teachers’ work engagement and students’ academic achievement, such that this relationship is strong for high (vs. low) self-esteem.

## Results

### Preliminary Analyses

Table 1 displays the means and standard deviations of correlations among variables examined in this study. Teacher EI is positively correlated with student academic achievement, providing preliminary evidence for H1. Teacher work engagement is positively correlated with teacher EI and student academic achievement, offering preliminary evidence for H2. As these variables except for academic achievement were measured by self-report scales, common-method bias was checked using Harman single-factor testing (Podsakoff and Organ, 1986). The testing yielded 11 factors with eigenvalues higher than one and the first factor only accounted for 20.216% of the total variance, so common-method bias is not a salient issue in this study.

**Table 1:** Descriptive statistics and correlations (N = 365).

Variable	M	SD	1	2	3	4
1. Emotional intelligence	4.859	1.018	–			
2. Work engagement	4.728	1.054	0.277^**^	–		
3. Self-efficacy	6.650	1.262	0.239^**^	0.433^**^	–	
4. Academic achievement	3.622	1.141	0.342^**^	0.502^**^	0.403^**^	–

** *p < 0.01*.

Table 2 shows the results of the total effect of teacher EI on student academic achievement. The fit goodness of the total effect model is acceptable: *χ*^2^ = 252.354, df = 243, *χ*^2^/df = 1.038, CFI = 0.994, TLI = 0.993, RMSEA = 0.010 (Hu and Bentler, 1999). H1 proposes that teacher EI is positively related to student academic achievement. As is shown in Table 2, teacher EI can positively predict student academic achievement (*b* = 0.572, *p* < 0.001). Therefore, H1 is supported.

**Table 2:** Total effect of teacher EI on student academic achievement.

Predictors	*B*	*SE*	*value of p*
**Control variables**			
Teacher gender	−0.108	0.110	0.323
Teacher age	−0.140	0.066	0.033
Teaching experience	0.145	0.048	0.003
Teacher education level	0.141	0.100	0.158
Class gender ratio	−0.371	0.955	0.698
Grade_7th	−0.325	0.137	0.017
Grade_8th	−0.241	0.138	0.080
AFI	0.156	0.049	0.001
**Independent variable**			
Teacher emotional intelligence	0.572	0.108	<0.001
R-square	0.224		

*B, unstandardized coefficient; SE, standard error; and grade was dummy coded (1 = 7th, 2 = 8th, and 3 = 9th)*.

### Mediating Effect Analysis

Table 3 shows the results of the mediating effect of teacher work engagement. The fit goodness of the total effect model is acceptable: *χ*^2^ = 849.355, df = 733, *χ*^2^/df = 1.159, CFI = 0.973, TLI = 0.971, RMSEA = 0.021 (Hu and Bentler, 1999). H2 proposes that teacher work engagement mediates the relationship between teacher EI and student academic achievement. As is shown in Table 3, teacher EI can positively predict teacher work engagement (*b* = 0.452, *p* < 0.001), and also, teacher work engagement can positively predict student academic achievement (*b* = 0.580, *p* < 0.001). Furthermore, the bootstrapping 95% CI for the mediating effect [*b* = 0.262, 95% CI = (0.156, 0.456)] does not contain zero, indicating that the examined mediating effect is statistically significant (Zhao et al., 2010). Thereby, H2 is supported. As the direct effect of teacher EI on student academic performance is still significant (*b* = 0.312, *p* = 0.001), teacher work engagement partially mediates the positive association between teacher EI and student academic performance.

**Table 3:** Mediating effect of teacher work engagement.

Predictors	Teacher work engagement			Student academic achievement		
	*B*	*SE*	*value of p*	*B*	*SE*	*value of p*
**Control variables**						
Teacher gender	−0.101	0.116	0.386	−0.050	0.100	0.616
Teacher age	−0.068	0.082	0.409	−0.101	0.067	0.132
Teaching experience	0.014	0.060	0.813	0.137	0.051	0.007
Teacher education level	0.074	0.114	0.514	0.097	0.091	0.285
Class gender ratio	−0.816	0.917	0.374	0.103	0.894	0.908
Grade_7th	−0.373	0.153	0.015	−0.109	0.130	0.403
Grade_8th	−0.322	0.156	0.039	−0.054	0.131	0.683
AFI	0.034	0.048	0.488	0.137	0.049	0.005
**Independent variable**						
Teacher emotional intelligence	0.452	0.115	<0.001	0.312	0.090	0.001
**Mediator**						
Teacher work engagement				0.580	0.085	<0.001
R-square	0.178			0.415		

*B, unstandardized coefficient; SE, standard error; and grade was dummy coded (1 = 7th, 2 = 8th, and 3 = 9th)*.

### Moderated Mediation Effect Analysis

Table 4 presents the results of the moderated mediation model. Since the LMS method do not provide traditional model fit indices, we followed the procedures recommended by Maslowsky et al. (2015) to access the fit goodness of the moderated mediation model. First, we ran a null model which excludes the latent interaction term (i.e., Teacher work engagement × Teacher self-efficacy), the result showed that this null model fits well (*χ*^2^ = 1509.717, df = 1,274, *χ*^2^/df = 1.185, CFI = 0.960, TLI = 0.958, RMSEA = 0.023). Second, we used log-likelihood ratio test to evaluate whether the model fit of the full model (i.e., the moderated mediation model including the latent interaction term) is significantly better than that of the null model. As indicated by the results of the log-likelihood ratio test {*χ*^2^ = −2[(−30175.443)–(−30171.076)] = 8.734, df = 1, *p* = 0.003}, the model fit of the full model is significantly better than that of the null model. Therefore, we can conclude that the moderated mediation model is also a well-fitted model (Maslowsky et al., 2015).

**Table 4:** Mediating effect of teacher work engagement moderated by teacher self-efficacy.

Predictors	Teacher work engagement			Student academic achievement		
	*B*	*SE*	*value of p*	*B*	*SE*	*value of p*
**Control variables**						
Teacher gender	−0.097	0.111	0.380	−0.028	0.097	0.773
Teacher age	−0.071	0.079	0.373	−0.113	0.064	0.080
Teaching experience	0.010	0.059	0.863	0.136	0.048	0.005
Teacher education level	0.069	0.111	0.531	0.051	0.087	0.561
Class gender ratio	−0.725	0.771	0.347	0.092	0.891	0.917
Grade_7th	−0.374	0.150	0.013	−0.089	0.126	0.483
Grade_8th	−0.305	0.153	0.046	0.016	0.128	0.902
AFI	0.026	0.047	0.578	0.123	0.047	0.008
**Independent variable**						
Teacher emotional intelligence	0.527	0.137	<0.001	0.235	0.100	0.018
**Mediator**						
Teacher work engagement				0.483	0.083	<0.001
**Moderator**						
Teacher self-efficacy				0.388	0.119	0.001
**Interaction term**						
Teacher work engagement **×** Teacher self-efficacy				0.162	0.053	0.002
R-square	0.222			0.436		

*B, unstandardized coefficient; SE, standard error; and grade was dummy coded (1 = 7th, 2 = 8th, and 3 = 9th)*.

**Figure 1:**
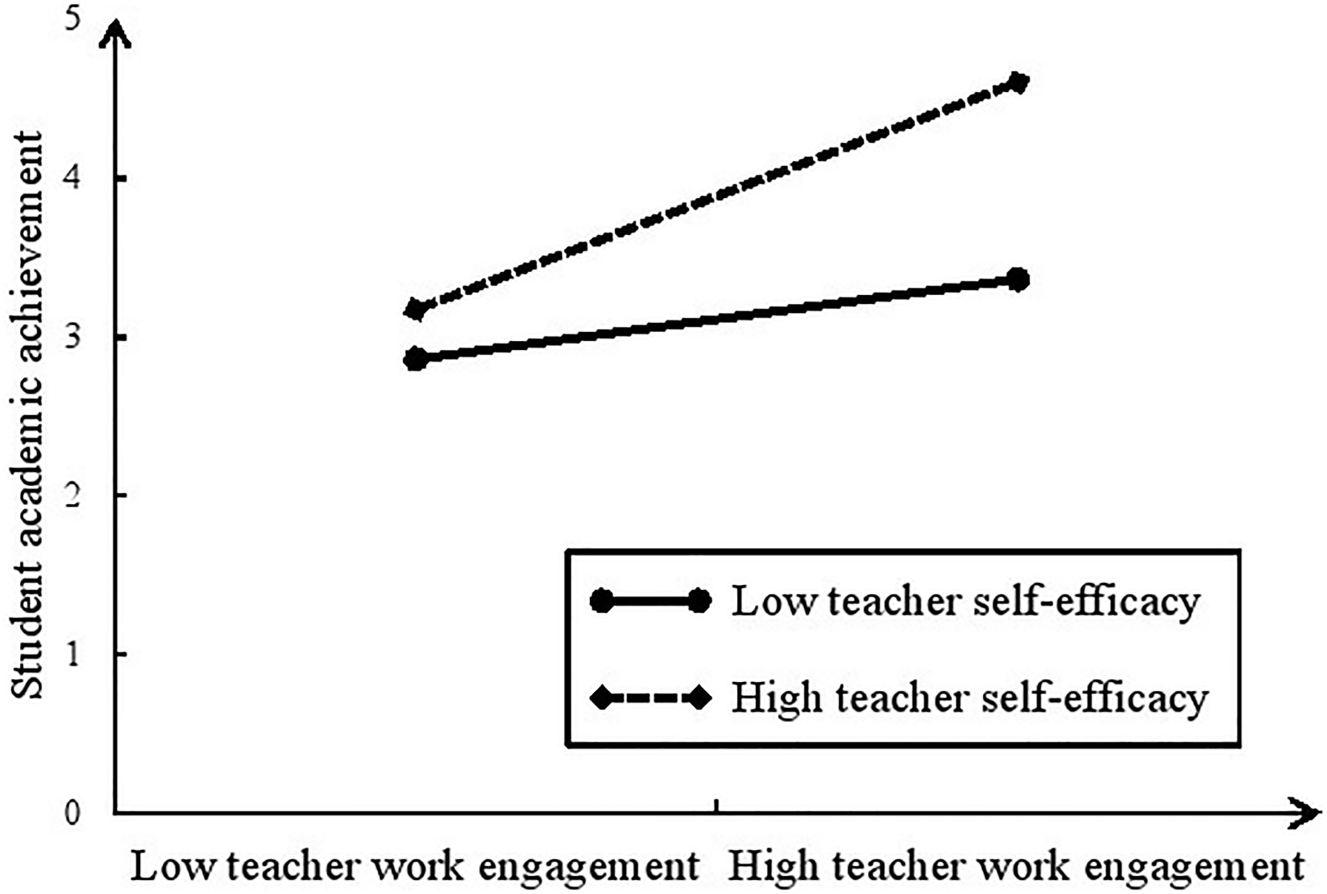
The moderating effect of teacher self-efficacy.

As is shown in Table 4, the path coefficient of the interaction term is significant and positive (*b* = 0.162, *p* = 0.002), indicating that the moderating effect of teacher self-efficacy on the association between teacher work engagement and student academic achievement is significant. Simple slope test was further conducted and the result showed that as: when teacher self-efficacy is low (M-SD), the path coefficient from teacher work engagement to student academic achievement is 0.326, but when teacher self-efficacy is high (M-SD), the path coefficient from teacher work engagement to student academic achievement is 0.641. The difference between the two coefficients is also significant (*diff* = 0.315, *p* = 0.004). Consequently, the relationship between teacher work engagement and student academic achievement is stronger for high (vs. low) teacher self-efficacy (see Figure 1), supporting H3. We also tested whether the mediating effect of teacher work engagement is moderated by teacher self-efficacy, and the results showed that the index of the moderated mediation is significant [index = 0.085, bootstrapping 95% CI = (0.032, 0.174) excluding zero]. Hence, teacher self-efficacy moderates the mediating effect of teacher work engagement.

## Discussion

### Main Findings

The present study used a moderated mediation to examine whether teacher work engagement would mediate the link between teacher EI and student academic achievement, and whether teacher self-efficacy would moderate the relationship between teacher work engagement and student academic achievement. Overall, our findings supported our hypotheses.

Consistent with our hypothesis, this study showed that teacher EI, as an important personal resource, could be a significant factor for students’ academic achievement. This finding does not support the results of such previous research performed by Koifman (1998), which found that teacher EI did not affect student achievement. This difference may be culturally related. Zhang and Zhu (2008) claimed that each culture has its unique emotional patterns, which have different meanings and effects on its members. In general, people from collectivist cultures perceive the self as a communal, relational entity that is connected to others. In the Chinese educational context, teachers play a dominant role in the classroom. Chinese students are more likely than Western students to have dependent relationships with their teachers.

Another major finding of this study was that teacher work engagement partially mediates the positive association between teacher EI and student academic achievement as the mediation model verification shows. Although quite a number of research has supported the relationship between teacher EI and work engagement (Mérida-López et al., 2017; D’Amico et al., 2020), as well as teacher work engagement and student academic achievement (Basikin, 2007; Addimando, 2019), to our knowledge, this study is the first to explore the mediating role of teacher work engagement between teacher emotional intelligence and student academic achievement. Consistent with our hypothesis, teacher EI could predict student academic achievement through the indirect effect of teacher work engagement. In other words, emotional intelligence can help teachers reduce burnout and thus become more engaged in the classroom, which in turn will improve student academic achievement.

Our findings confirmed that teacher self-efficacy played a moderating role in the influence of teacher work engagement on student academic achievement. Prior studies have revealed that teachers with a strong sense of efficacy exhibit greater levels of engagement (Skaalvik and Skaalvik, 2014; Granziera and Perera, 2019) and have a more positive impact on students’ academic learning (Tschannen-Moran and Woolfolk Hoy, 2001; Kim and Seo, 2018). However, those studies did not explore the moderating role of teacher self-efficacy between teacher work engagement and student academic achievement. This study found that teachers with high self-efficacy had a more significant positive impact on the relationship between teacher work engagement and student academic achievement than teachers with low self-efficacy. Our findings also support the opinion that teachers with higher levels of self-efficacy appear to view teaching challenges as a controllable factor and are more likely to use innovative teaching methods in order to help their students succeed (Basikin, 2007).

### Limitations

The limitations of the current study should be mentioned. First, only students and teachers from Chinese schools were assessed, which is a small number, which may affect the representativeness of the sample. A large sample of students and teachers from different countries, different ages, and different cultures will more accurately reveal the influence of teacher EI on student academic achievement. Second, this study only focuses on the influence of teacher EI, self-efficacy, and work engagement on student academic achievement, and does not involve student EI, self-efficacy, and work engagement. These factors also have an important impact on student academic achievement, and their influencing mechanism and joint effect are worth further study. Third, there may be other mediators, moderating variables, and relationship models. Individual emotion, personality characteristics, school atmosphere, family atmosphere, and peers may play a mediating or moderating role in the relationship between teacher EI and student academic achievement. This study establishes a moderated mediation model between teacher self-efficacy and work engagement about student academic achievement but does not exclude the possibility of multiple and mediated moderating models.

## Conclusion

In sum, the present study may be the first study to investigate the correlation between teacher EI and student academic achievement by examining a moderated mediation model. It reveals that the relationship between teacher EI and student academic achievement could be mediated by teacher work engagement. Besides, the link between teacher work engagement and student achievement is moderated by teacher self-efficacy. Our study hopes that EI training will be considered as part of the training program for pre-service teachers. In addition, schools can provide EI training to teachers who face difficulties with classroom control or expect to build good relationships with students.

## Data Availability Statement

The raw data supporting the conclusions of this article will be made available by the authors, without undue reservation.

## Author Contributions

The author confirms being the sole contributor of this work and has approved it for publication.

## Funding

The completion of this article has been supported by the project of Hubei University of Arts and Science (XK2020021).

## Conflict of Interest

The author declares that the research was conducted in the absence of any commercial or financial relationships that could be construed as a potential conflict of interest.

## Publisher’s Note

All claims expressed in this article are solely those of the authors and do not necessarily represent those of their affiliated organizations, or those of the publisher, the editors and the reviewers. Any product that may be evaluated in this article, or claim that may be made by its manufacturer, is not guaranteed or endorsed by the publisher.

## References

[ref1] Addimando L. (2019). The effect of positive working conditions on work engagement and teaching classroom practices: a large cross-sectional study in Switzerland. Front. Psychol. 10:02129. doi: 10.3389/fpsyg.2019.02129 PMC6763685 31616341

[ref2] Alam A. Ahmad M. (2018). The role of teachers’ emotional intelligence in enhancing student achievement. J. Asia Bus. Stud. 12, 31–43. doi: 10.1108/JABS-08-2015-0134

[ref3] Bakker A. B. Demerouti E. (2017). Job demands–resources theory: taking stock and looking forward. J. Occup. Health Psychol. 22, 273–285. doi: 10.1037/ocp0000056, PMID: 27732008

[ref4] Bandura A. (1997). Self-Efficacy: The Exercise of Control. New York, NY: W. H. Freeman.

[ref5] Bar-On R. (2010). Emotional intelligence: An integral part of positive psychology. S. Afr. J. Psychol. 40, 54–62. doi: 10.1177/008124631004000106

[ref6] Bar-On R. E. Parker J. D. (2000). The Handbook of Emotional Intelligence: Theory, Development, Assessment, and Application at Home, School, and in the Workplace. The Handbook of Emotional Intelligence. San Francisco, CA: Jossey-Bass.

[ref7] Basikin (2007). “Vigor, dedication and absorption: work engagement among secondary school English teachers in Indonesia.” in 2007 AARE International Conference, 27–28 November 2007, University of Notre Dame, Australia, Fremantle, Perth.

[ref8] Berridge K. C. (2018). Evolving concepts of emotion and motivation. Front. Psychol. 9:1647. doi: 10.3389/fpsyg.2018.01647, PMID: 30245654 PMC6137142

[ref9] Brackett M. A. Mayer J. D. Warner R. M. (2004). Emotional intelligence and its relation to everyday behaviour. Personal. Individ. Differ. 36, 1387–1402. doi: 10.1016/S0191-8869(03)00236-8

[ref10] Chan D. W. (2004). Perceived emotional intelligence and self-efficacy among Chinese secondary school teachers in Hong Kong. Personal. Individ. Differ. 36, 1781–1795. doi: 10.1016/j.paid.2003.07.007

[ref11] Cheng C. H. Wang Y. C. Liu W. X. (2019). Exploring the related factors in students’ academic achievement for the sustainable education of rural areas. Sustainability. 11:5974. doi: 10.3390/su11215974

[ref12] Cheung G. W. Lau R. S. (2017). Accuracy of parameter estimates and confidence intervals in moderated mediation models: A comparison of regression and latent moderated structural equations. Organ. Res. Methods 20, 746–769. doi: 10.1177/1094428115595869

[ref13] Christian M. S. Garza A. S. Slaughter J. E. (2011). Work engagement: A quantitative review and test of its relations with task and contextual performance. Pers. Psychol. 64, 89–136. doi: 10.1111/j.1744-6570.2010.01203.x

[ref14] Curci A. Lanciano T. Soleti E. (2014). Emotions in the classroom: The role of teachers’ emotional intelligence ability in predicting students’ achievement. Am. J. Psychol. 127, 431–445. doi: 10.5406/amerjpsyc.127.4.0431, PMID: 25603580

[ref15] D’Amico A. Geraci A. Tarantino C. (2020). The relationship between perceived emotional intelligence, work engagement, job satisfaction, and burnout in Italian school teachers: an exploratory study. Psihologijske teme. 29, 63–84. doi: 10.31820/pt.29.1.4

[ref16] Davis S. K. Humphrey N. (2014). Ability versus trait emotional intelligence: dual influences on adolescent psychological adaptation. J. Individ. Dif. 35, 54–62. doi: 10.1027/1614-0001/a000127

[ref17] Davis S. K. Nichols R. (2016). Does emotional intelligence have a “dark” side? A review of the literature. Front. Psychol. 7:1316. doi: 10.3389/fpsyg.2016.01316, PMID: 27625627 PMC5003940

[ref18] Durlak J. A. Weissberg R. P. Dymnicki A. B. Taylor R. D. Schellinger K. B. (2011). The impact of enhancing students’ social and emotional learning: a meta-analysis of school-based universal interventions. Child Dev. 82, 405–432. doi: 10.1111/j.1467-8624.2010.01564.x, PMID: 21291449

[ref19] Eisma M. C. Stroebe M. S. (2021). Emotion regulatory strategies in complicated grief: A systematic review. Behav. Ther. 52, 234–249. doi: 10.1016/j.beth.2020.04.004, PMID: 33483120

[ref20] Extremera N. Fernández-Berrocal P. (2013). Inteligencia emocional en adolescentes. Padres y Maestros 352, 34–39. doi: 10.14422/pym.v0i352.1170

[ref21] Frenzel A. C. (2014). “Teacher emotions,” in International Handbook of Emotions in Education. eds. Linnenbrink-Garcia E. A. Pekrun R. (New York, NY: Routledge), 494–519.

[ref22] Granziera H. Perera H. N. (2019). Relations among teachers’ self-efficacy beliefs, engagement, and work satisfaction: a social cognitive view. Contemp. Educ. Psychol. 58, 75–84. doi: 10.1016/j.cedpsych.2019.02.003

[ref23] Hayes A. F. (2015). An index and test of linear moderated mediation. Multivar. Behav. Res. 50, 1–22. doi: 10.1080/00273171.2014.962683, PMID: 26609740

[ref24] Hu L. T. Bentler P. M. (1999). Cutoff criteria for fit indexes in covariance structure analysis: conventional criteria versus new alternatives. Struct. Equ. Model. Multidiscip. J. 6, 1–55. doi: 10.1080/10705519909540118

[ref25] Kim K. R. Seo E. H. (2018). The relationship between teacher efficacy and students’ academic achievement: A meta-analysis. Soc. Behav. Personal. Int. J. 46, 529–540. doi: 10.2224/sbp.6554

[ref26] Klassen R. M. Tze V. M. C. Betts S. M. Gordon K. A. (2011). Teacher Efficacy Research 1998–2009: Signs of Progress or Unfulfilled Promise? Educ Psychol Rev. 23, 21–43. doi: 10.1007/s10648-010-9141-8

[ref27] Klassen R. M. Yerdelen S. Durksen T. L. (2013). Measuring teacher engagement: development of the engaged teachers scale (ETS). Frontline Learn. Res. 1, 33–52. doi: 10.14786/flr.v1i2.44

[ref28] Koifman R. (1998). The Relationship between EQ, IQ, and Creativity. Unpublished manuscript, York University, Toronto.

[ref30] Lu J. M. Chen N. Q. Xu L. Chen Y. X. Wu J. (2016). A survey of contemporary college students’ emotional intelligence in China. J. Psychol. Sci. 39, 1302–1309. doi: 10.16719/j.cnki.1671-6981.20160604

[ref31] Maamari B. E. Majdalani J. F. (2019). The effect of highly emotionally intelligent teachers on their students’ satisfaction. Int. J. Educ. Manag. 33, 179–193. doi: 10.1108/IJEM-11-2017-0338

[ref32] Maslowsky J. Jager J. Hemken D. (2015). Estimating and interpreting latent variable interactions: A tutorial for applying the latent moderated structural equations method. Int. J. Behav. Dev. 39, 87–96. doi: 10.1177/0165025414552301, PMID: 26478643 PMC4606468

[ref33] Mayer J. D. Salovey P. Caruso D. R. (2004). Emotional intelligence: theory, findings, and implications. Psychol. Inq. 15, 197–215. doi: 10.1207/s15327965pli1503_02

[ref34] Mérida-López S. Extremera N. (2017). Emotional intelligence and teacher burnout: A systematic review. Int. J. Educ. Res. 85, 121–130. doi: 10.1016/j.ijer.2017.07.006

[ref35] Mérida-López S. Extremera N. Rey L. (2017). Contributions of work-related stress and emotional intelligence to teacher engagement: additive and interactive effects. Int. J. Environ. Res. Public Health 14, 1–16. doi: 10.3390/ijerph14101156, PMID: 28961218 PMC5664657

[ref36] Miao C. Humphrey R. H. Qian S. (2017). A meta-analysis of emotional intelligence and work attitudes. J. Occup. Organ. Psychol. 90, 177–202. doi: 10.1111/joop.12167

[ref37] Minghui L. Lei H. Xiaomeng C. Potměšilc M. (2018). Teacher efficacy, work engagement, and social support among Chinese special education school teachers. Front. Psychol. 9:648. doi: 10.3389/fpsyg.2018.00648, PMID: 29867634 PMC5949842

[ref38] Moafian F. Ghanizadeh A. (2009). The relationship between Iranian EFL teachers’ emotional intelligence and their self-efficacy in language institutes. System 37, 708–718. doi: 10.1016/j.system.2009.09.014

[ref39] Muthén L. K. Muthén B. O. (2015). Mplus User’s Guide. 7th Edn. Los Angeles, CA: Muthén and Muthén.

[ref01] Naderi Anari N. (2012). Teachers: emotional intelligence, job satisfaction, and organizational commitment. J. Workplace Learn. 24, 256–269. doi: 10.1108/13665621211223379

[ref40] O’Boyle E. H. Jr. Humphrey R. H. Pollack J. M. Hawver T. H. Story P. A. (2011). The relation between emotional intelligence and job performance: A meta-analysis. J. Organ. Behav. 32, 788–818. doi: 10.1002/job.714

[ref41] Palomera R. Fernández-Berrocal P. Brackett M. A. (2008). Emotional intelligence as a basic competency in pre-service teacher training: some evidence. Electron. J. Res. Educ. Psychol. 6, 437–454. doi: 10.25115/ejrep.v6i15.1292

[ref42] Perera H. N. Calkins C. Part R. (2019). Teacher self-efficacy profiles: determinants, outcomes, and generalizability across teaching level. Contemp. Educ. Psychol. 58, 186–203. doi: 10.1016/j.cedpsych.2019.02.006

[ref43] Pishghadam R. Sahebjam S. (2012). Personality and emotional intelligence in teacher burnout. Span. J. Psychol. 15, 227–236. doi: 10.5209/rev_SJOP.2012.v15.n1.37314, PMID: 22379712

[ref44] Platsidou M. (2010). Trait emotional intelligence of Greek special education teachers in relation to burnout and job satisfaction. Sch. Psychol. Int. 31, 60–76. doi: 10.1177/0143034309360436

[ref45] Podsakoff P. M. Organ D. W. (1986). Self-reports in organizational research: problems and prospects. J. Manag. 12, 531–544. doi: 10.1177/014920638601200408

[ref46] Preacher K. J. Hayes A. F. (2008). Asymptotic and resampling strategies for assessing and comparing indirect effects in multiple mediator models. Behav. Res. Methods 40, 879–891. doi: 10.3758/BRM.40.3.879, PMID: 18697684

[ref47] Salovey P. J. Mayer D. (1990). Emotional intelligence. Imag. Cogn. Persy. 9, 267–298. doi: 10.2190/DUGG-P24E-52WK-6CDG

[ref48] Schaufeli W. B. Salanova M. González-Romá V. Bakker A. B. (2002). The measurement of engagement and burnout: A two sample confirmatory factor analytic approach. J. Happiness Stud. 3, 71–92. doi: 10.1023/A:1015630930326

[ref49] Schonert-Reichl K. A. (2017). Social and emotional learning and teachers. Futur. Child. 27, 137–155. doi: 10.1353/foc.2017.0007

[ref51] Simbula S. Guglielmi D. Schaufeli W. B. (2011). A three-wave study of job resources, self-efficacy, and work engagement among Italian schoolteachers. Eur. J. Work Organ. Psy. 20, 285–304. doi: 10.1080/13594320903513916

[ref52] Singh I. Jha A. (2012). Teacher effectiveness in relation to emotional intelligence among medical and engineering faculty members. Eur. J. Psychol. 8, 667–685. doi: 10.5964/ejop.v8i4.483

[ref53] Skaalvik E. M. Skaalvik S. (2014). Teacher self-efficacy and perceived autonomy: relations with teacher engagement, job satisfaction, and emotional exhaustion. Psychol. Rep. 114, 68–77. doi: 10.2466/14.02.PR0.114k14w0, PMID: 24765710

[ref54] Tschannen-Moran M. Woolfolk Hoy A. (2001). Teacher efficacy: capturing and elusive construct. Teach. Teach. Educ. 17, 783–805. doi: 10.1016/S0742-051X(01)00036-1

[ref55] Valente S. Veiga-Branco A. Rebelo H. Lourenço A. A. Cristóvão A. M. (2020). The relationship between emotional intelligence ability and teacher efficacy. Univ. J. Educ. Res. 8, 916–923. doi: 10.13189/ujer.2020.080324

[ref56] Wang H. Wu S. Wang W. Wei C. (2021). Emotional intelligence and prosocial behavior in college students: A moderated mediation analysis. Front. Psychol. 12:713227. doi: 10.3389/fpsyg.2021.713227, PMID: 34552535 PMC8450319

[ref57] Welmilla I. (2020). Students’ perspective on the emotional intelligence of teachers on student engagement. Int. Bus. Res. 13, 30–30. doi: 10.5539/ibr.v13n4p30

[ref58] Wong C. S. Law K. S. (2002). The effects of leader and follower emotional intelligence on performance and attitude: An exploratory study. Leadersh. Q. 13, 243–274. doi: 10.1037/t07398-000

[ref59] Wu Y. Lian K. Hong P. Liu S. Lin R. M. Lian R. (2019). Teachers’ emotional intelligence and self-efficacy: mediating role of teaching performance. Soc. Behav. Personal. Int. J. 47, 1–10. doi: 10.2224/sbp.7869

[ref60] Yin H. (2012). Adaptation and validation of the teacher emotional labour strategy scale in China. Educ. Psychol. 32, 451–465. doi: 10.1080/01443410.2012.674488

[ref61] Zhang Q. Zhu W. (2008). Exploring emotion in teaching: emotional labor, burnout, and satisfaction in Chinese higher education. Commun. Educ. 57, 105–122. doi: 10.1080/03634520701586310

[ref62] Zhao X. Lynch J. G. Jr. Chen Q. (2010). Reconsidering Baron and Kenny: myths and truths about mediation analysis. J. Consum. Res. 37, 197–206. doi: 10.1086/651257

